# Inhibition of SOC/Ca^2+^/NFAT pathway is involved in the anti-proliferative effect of sildenafil on pulmonary artery smooth muscle cells

**DOI:** 10.1186/1465-9921-10-123

**Published:** 2009-12-11

**Authors:** Cong Wang, Ji-Feng Li, Lan Zhao, Jie Liu, Jun Wan, Yue Xiu Wang, Jun Wang, Chen Wang

**Affiliations:** 1 Beijing Institute of Respiratory Medicine, Beijing Chao-Yang Hospital, Capital Medical University, Beijing, PR China; 2 Department of Physiology, Capital Medical University, Beijing, PR China; 3 Department of Respiratory Disease, Capital Medical University, Beijing, PR China; 4 Experimental Medicine and Toxicology, Imperial College London, Hammersmith Hospital, UK

## Abstract

**Background:**

Sildenafil, a potent phosphodiesterase type 5 (PDE5) inhibitor, has been proposed as a treatment for pulmonary arterial hypertension (PAH). The mechanism of its anti-proliferative effect on pulmonary artery smooth muscle cells (PASMC) is unclear. Nuclear translocation of nuclear factor of activated T-cells (NFAT) is thought to be involved in PASMC proliferation and PAH. Increase in cytosolic free [Ca^2+^] ([Ca^2+^]_i_) is a prerequisite for NFAT nuclear translocation. Elevated [Ca^2+^]_i _in PASMC of PAH patients has been demonstrated through up-regulation of store-operated Ca^2+ ^channels (SOC) which is encoded by the transient receptor potential (TRP) channel protein. Thus we investigated if: 1) up-regulation of TRPC1 channel expression which induces enhancement of SOC-mediated Ca^2+ ^influx and increase in [Ca^2+^]_i _is involved in hypoxia-induced PASMC proliferation; 2) hypoxia-induced promotion of [Ca^2+^]_i _leads to nuclear translocation of NFAT and regulates PASMC proliferation and TRPC1 expression; 3) the anti-proliferative effect of sildenafil is mediated by inhibition of this SOC/Ca^2+^/NFAT pathway.

**Methods:**

Human PASMC were cultured under hypoxia (3% O_2_) with or without sildenafil treatment for 72 h. Cell number and cell viability were determined with a hemocytometer and MTT assay respectively. [Ca^2+^]_i _was measured with a dynamic digital Ca^2+ ^imaging system by loading PASMC with fura 2-AM. TRPC1 mRNA and protein level were detected by RT-PCR and Western blotting respectively. Nuclear translocation of NFAT was determined by immunofluoresence microscopy.

**Results:**

Hypoxia induced PASMC proliferation with increases in basal [Ca^2+^]_i _and Ca^2+ ^entry via SOC (SOCE). These were accompanied by up-regulation of TRPC1 gene and protein expression in PASMC. NFAT nuclear translocation was significantly enhanced by hypoxia, which was dependent on SOCE and sensitive to SOC inhibitor SKF96365 (SKF), as well as cGMP analogue, 8-brom-cGMP. Hypoxia-induced PASMC proliferation and TRPC1 up-regulation were inhibited by SKF and NFAT blocker (VIVIT and Cyclosporin A). Sildenafil treatment ameliorated hypoxia-induced PASMC proliferation and attenuated hypoxia-induced enhancement of basal [Ca^2+^]_i_, SOCE, up-regulation of TRPC1 expression, and NFAT nuclear translocation.

**Conclusion:**

The SOC/Ca^2+^/NFAT pathway is, at least in part, a downstream mediator for the anti-proliferative effect of sildenafil, and may have therapeutic potential for PAH treatment.

## Background

Pulmonary arterial hypertension (PAH) is a progressive disease characterized by a sustained increase in pulmonary arterial pressure and vascular remodeling. A few molecular mechanisms such as prostacyclin, nitric oxide (NO)/cyclic guanosine monophosphate (cGMP) and endothelin pathways have been shown of pathological importance and involved in the abnormal proliferation and contraction of pulmonary artery smooth muscle cells (PASMC) in PAH patients. Therapies developed towards these targets, such as prostacyclin analogs, endothelin-1 receptor antagonists and phosphodiesterase type-5 (PDE5) inhibitors [1], have been shown of clinical benefit. One PDE5 inhibitor, sildenafil has been demonstrated to inhibit pulmonary hypertension secondary to chronic hypoxia in rats [2]. Long-term adjunctive treatment with oral sildenafil improved New York Heart Association Class and 6-min walk distance in PAH patients [3]. Sildenafil, through inhibition of cGMP breakdown by PDE5 in PASMC, exerts its NO-dependent cGMP-mediated pulmonary vasodilatory effects. Recent evidence indicates that NO/cGMP signaling is not attenuated but up-regulated in a hypoxic mouse model of PAH, and sildenafil merely acts as an effective pulmonary vasodilator by further augmenting this pathway [4]. Furthermore, the anti-proliferative properties of sildenafil may operate through other signaling molecules in addition to the NO/cGMP axis by targeting PKG/PKA [5].

Nuclear factor of activated T-cells (NFAT) is a signal integrator of Ca^2+ ^signal and other signaling pathways through induction of a specific genetic program, and it has been proposed to be involved in PAH pathogenesis. The Ca^2+^/NFAT pathway plays an important part in the cell proliferation including osteoblasts [6], pancreatic beta cells [7], human myometrial vascular smooth muscle cells [8], rat aortic myocytes [9], rat cardiac myocytes and fibroblasts [10], and skeletal muscle reserve cells [11]. Chronic hypoxia induces NFAT transcriptional activity increase and NFATc3 nuclear translocation in mouse pulmonary arteries [12]. Increased NFATc2 protein level associated with a more nuclear localization, was observed in PASMC isolated from idiopathic PAH patients, suggesting enhanced NFAT activation might contribute to vascular remodeling in this disease [13].

Calcineurin, a calcium- and calmodulin-dependent phosphatase, is known to be a mediator of NFAT signaling, which induces NFAT proteins de-phosphorylation and nuclear translocation [14,15]. Calcineurin phosphatase activity is critically dependent on [Ca^2+^]_i_. Ca^2+ ^influx is the important determinant of NFAT activity in skeletal muscle cells and smooth muscle cells [15].

Two main types of calcium channels in the human PASMC membrane mediate Ca^2+ ^influx: voltage-dependent calcium channels (VDCC) and voltage-independent calcium channels (VICC). The latter include store-operated channels (SOC) and receptor-operated channels (ROC). When humoral factors such as endothelin-1 (ET-1) bind G-protein-coupled receptors (GPCR) or receptor tyrosine kinase (RTK), they will activate phospholipase-C (PLC) to produce inositol 1,4,5-trisphosphate (IP3) and diacylglycerol (DAG). IP3-induced Ca^2+ ^release from the endoplasmic reticulum (ER) produces a transient increase in [Ca^2+^]_i_. Subsequently, the depletion of intracellular Ca^2+ ^stores triggers a sustained Ca^2+ ^flux called capacitive calcium entry (CCE). Ca^2+ ^entry via SOC (SOCE) in the membrane caused by ER depletion is the dominated component of CCE [16]. Ca^2+ ^influx via SOC appears to be a determinant in maintaining a sustained increase in [Ca^2+^]_i _and regulation of vascular tone and arterial wall structure [17]. Elevated influx of Ca^2+ ^via SOC in PASMC had been observed in animal models and patients of PAH [18,19].

Native SOC are believed to be encoded by a novel family of transient receptor potential (TRP) channels, a large superfamily of channels permeable to Ca^2+^. Members of canonical transient receptor potential channels (TRPC) have been identified in PASMC. The involvement of TRPC1 in SOC in human PASMC has been demonstrated and it contributes to the development of pulmonary vascular remodeling in PAH patients [17,20,21].

Thus, we hypothesized that hypoxia-induced PASMC proliferation involves up-regulation of TRPC1 expression, which in turn resulted in the enhancement of SOCE and elevation of [Ca^2+^]_i_. The promoted [Ca^2+^]_i _leads to increased calcineurin phosphatase activity, which induces nuclear translocation of NFAT. NFAT activation in PASMC could regulate multiple gene transcriptions including TRPC1 gene which positively reinforce NFAT activation and cell proliferation. The SOC/Ca^2+^/NFAT pathway may be a downstream mediator for the anti-proliferative effect of sildenafil.

## Methods

### Cell culture

Human PASMC from normal human subjects were purchased from Cascade Biologics Incorporated (Portland, OR, USA). PASMC (Passages 4-8) were cultured in smooth muscle growth medium (SMGM), which consisted of smooth muscle basal medium (SMBM; M231; Cascade Biologics) and smooth muscle growth supplement (SMGS; Cascade Biologics). The final concentration of SMGS contained 4.9% fetal bovine serum (FBS), 2 ng/mL basic fibroblast growth factor, 0.5 ng/mL epidermal growth factor, 5 ng/mL heparin, 5 mg/mL insulin and 0.2 mg/mL bovine serum albumin (BSA). Cells were maintained at 37°C in a humidified normoxia (21% O_2_, 5% CO_2_, 74% N_2_) and passaged after reaching 80-90% confluence. Cell growth was arrested by replacing SMGM with growth supplement-free SMBM for 24 h under normoxia [22]. For hypoxia experiments, growth-arrested cells were incubated with low-serum SMBM (2% FBS) under normoxia and hypoxia for 72 h, respectively.

### Determination of cell proliferation

Cell proliferation was quantified by cell counting with a hemocytometer or methyl thiazolyl tetrazolium (MTT) assay (Sigma-Aldrich, St. Louis, MO, USA). Briefly, PASMC were seeded in 24-well microplates at 1 × 10^4 ^cells/well. Cell number was determined with a hemocytometer using 0.45% trypan blue (Sigma-Aldrich, St Louis, MO, USA). For MTT assay, cells were plated into 96-well microplates at 5 × 10^3 ^cells/well and treated with different drugs for 72 h. After incubation, 20 μL of the MTT reagent was added to each well and the multi-well plates incubated in a humidified atmosphere for 4 h. The supernatant was removed and dimethyl sulfoxide (DMSO, Sigma-Aldrich, Shanghai, China) of 150 μL/well was added to the plates to solubilize the formazan salt crystals. Plates were incubated for 10 min on a swing bed at room temperature. Solubilized formazan products were quantified by spectrophotometry at 570 nm using an enzyme-linked immunosorbent assay (ELISA) reader (Bio-Rad, Japan). Data were expressed as percentage of control.

### Measurement of [Ca^2+^]_i_

[Ca^2+^]_i _in a single cell was measured using a Ca^2+^-sensitive fluorescent indicator fura 2-AM (Invitrogen, Carlsbad, CA, USA). Cells were loaded with 3 μM fura 2-AM for 30 min in the dark at room temperature. Fura 2-AM loaded cells were transferred to glass-bottomed culture dishes (MatTek Corporation, Ashland, MA, USA), fixed on a microscope stage, and perfused with physiological salt solution (PSS) for 30 min to remove extracellular fura 2-AM and to activate intracellular fura 2-AM into fura 2. The [Ca^2+^]_i _was measured using an xenon lamp (Lambda DG4, Sutter Instrument Company, Novato, CA, USA) equipped with a Nikon's Epi-fluorescence microscope (TE2000-U; Nikon, Tokyo, Japan) and band-pass filters for wavelengths of 340 nm and 380 nm. [Ca^2+^]_i _was based on the equation, [Ca^2+^]_i _= K_d _× (Sf2/Sb2) × (R-Rmin)/(Rmax-R) [Kd was assumed to be 224 nm, R was the fluorescence ratio at 340/380 nm, Sf2 and Sb2 were the ratio of free and bound forms of the dye. Rmin and Rmax were the 340 nm/380 nm ratios of full free and full bound][23]. Resting [Ca^2+^]_i_, cyclopiazonic acid (CPA; Sigma-Aldrich, Rehovot, Israel)-induced ER Ca^2+ ^release and SOCE upon changing perfusion from Ca^2+^-free PSS to 1.8 mM Ca^2+ ^PSS were measured in different groups. In most experiments, 5-10 cells were imaged in a single field, and a selected peripheral cytosolic area from each cell used for analysis.

### Reverse transcriptase-polymerase chain reaction (RT-PCR)

Total RNA was isolated from PASMC by using TRIzol reagent (Sigma-Aldrich. St. Louis, MO, USA) according to manufacturer's instructions. RNA was reverse-transcribed to synthesize first-strand cDNA. The specific primers were designed from coding regions of human TRPC1 (forward primer: 5'-CAAGATTTTGGAAAATTTCTTG-3', reverse primer: 5'-TTTGTCTTCATGATTTGCTAT-3'). The primers of β-actin (forward primer: 5'-GTGGGGCGCCCCAGGCACCA-3', reverse primer: 5'-CTTCCTTAATGTCACGCACGATTTC-3') were used as control for RNA integrity. PCR was done using an Icycler Thermal cycler (Bio-Rad, Hercules, CA, USA) under conditions described below. The PCR reaction mixture was denatured at 94°C (0.5 min), annealed at 55°C (0.5 min), and extended at 72°C (0.5 min) for 30 cycles. This was followed by a final extension at 72°C (5 min) to ensure complete product extension. Amplified products were separated by 1.5% agarose gel electrophoresis and stained with ethidium bromide. PCR product bands were visualized by ultraviolet light (Bio-Rad, Milan, Italy). Intensity values were measured by densitometric analysis with Quantitative One software (Bio-Rad, Milan, Italy), and normalized to the intensity values of β-actin for quantitative comparisons. PCR products were sequenced. The amplified production of TRPC1 and β-actin were 372 bp and 539 bp respectively. The ratio of normoxia group was regarded as 100%.

### Protein extraction and Western blotting

TRPC1 protein was detected using a standard Western blotting protocol. Briefly, adherent PASMC were harvested and 40 μg proteins from each sample of different groups separated by 8% sodium dodecyl sulfate-polyacrylamide gel electrophoresis (SDS-PAGE) at 80 V for 0.5 h, and at 120 V for 1.5 h. They were transferred onto a nitrocellulose membrane (Millipore, Billerica, MA, USA) at 100 V for 1.5 h at 4°C onto Western blotting apparatus (Bio-Rad, Hercules, CA, USA). The blocked membrane was incubated with primary antibody of TRPC1 (dilution, 1:1000; Alomone Laboratories, Jerusalem, Israel) and β-actin (dilution, 1:1000; Santa Cruz Biotechnology, Santa Cruz, CA, USA) overnight at 4°C. After incubation with horseradish peroxidase-conjugated secondary antibody (dilution, 1:2000; Beijing Zhongshan Golden Bridge Biological Technology Company, Beijing, China) for 1 h at room temperature, immunoblotting signals were visualized using Western Luminescent kit (Vigorous Biotechnology, Beijing, China). Results were quantified by densitometry, and the densities of immunoblotting were analyzed by scanning X-ray film with Quantitative One software. The value of the relative density of the TRPC1 band was normalized to the density of the β-actin band to represent the amount of TRPC1 protein. The ratio of normoxia group was regarded as 100%.

### Immunofluorescence microscopy

The human PASMC after 24 h starvation were cultured in 2% FBS under normoxia, hypoxia or hypoxia plus sildenafil or other drugs for 72 h respectively. After treatment, cells were fixed for 30 min at room temperature in 4% formaldehyde in Dulbecco's Phosphate-Buffered Saline (D-PBS), blocked with blocking solution (2% BSA in D-PBS) for 15 min and incubated with 0.2% Triton X-100 in blocking buffer for 30 min at room temperature. Cells were incubated with primary antibodies (NFATc3, sc-8321 Santa Cruz Biotechnology, Santa Cruz, CA, USA) for 1 h at room temperature and then fluorescent-conjugated secondary antibodies [Rhodamine (TRITC)-conjugated AffiniPure Goat Anti-mouse IgG, Beijing Zhongshan Golden Bridge Biological Technology Company, Beijing, China] for 30 min at room temperature. The nucleus was stained with Hoechest33258 (Sigma-Aldrich. St. Louis, MO, USA). Fluorescence was examined using a Leica laser scanning confocal microscope (TCS SP5, Leica, Wetzlar, Germany).

### Drugs and Reagents

PSS contained (in mM): 141 NaCl, 4.7 KCl, 1.8 CaCl_2_, 1.2 MgCl_2_, 10 HEPES, and 10 glucose, (pH 7.4). For Ca^2+^-free PSS, CaCl_2 _was replaced by equimolar MgCl_2 _and 1 mM EGTA added to chelate residual Ca^2+ ^[21]. CPA, fura 2-AM, SKF96365 (SKF; Sigma-Aldrich. St. Louis, MO, USA) and nifedipine (Sigma-Aldrich. St. Louis, MO, USA) were dissolved in DMSO to make stock solutions. Gadolinium chloride (GdCl_3_, Sigma-Aldrich. St. Louis, MO, USA), VIVIT (480401, Calbiochem, Darmstadt, Germany) and 8-brom-cGMP (Sigma-Aldrich. St. Louis, MO, USA) were dissolved in deionized water to form the stock solution. Cyclosporine A (1101, MBL International, Woburn, MA) was dissolved in ethanol to form the stock solution. MTT was dissolved in PBS to form stock solution. Sildenafil (Pfizer, Sandwich, Kent, UK) was dissolved in distilled water (pH 5.3) to make a stock solution of 1 mM.

### Statistical analysis

Data are mean ± SEM. At least six independent PASMC cultures were used. Comparison between groups of data was evaluated using the Student's unpaired *t*-test. For multiple comparisons, one-way analysis of variance (ANOVA) was used with a Bonferroni *post hoc *test (*P *< 0.05 was considered significant).

## Results

### Sildenafil inhibits hypoxia-induced human PASMC proliferation

**Figure 1:**
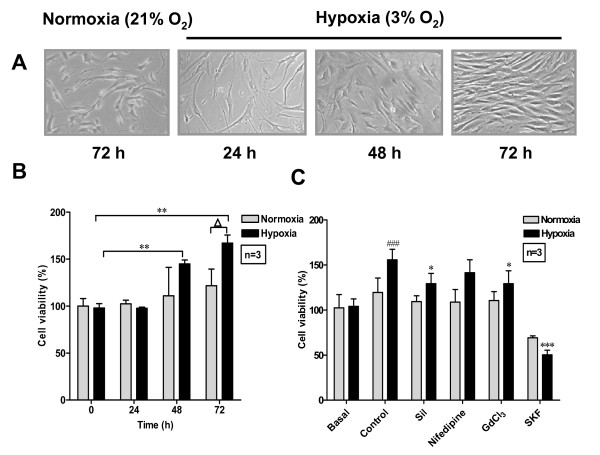
**Hypoxia-induced human PASMC proliferation and its dependence on SOC**. Human PASMC were cultured with SMBM (2% FBS) in normoxia or hypoxia for different time. A: Phase contrast image of cultured human PASMC (×200). B: Cell viability was determined by MTT. n = 3, **P < 0.01, Δ*P *< 0.05. C: Cell viability was determined before (Basal) and after 72 h incubation under normoxia and hypoxia without (Control) or with different agents: sildenafil (Sil 100 nM), nifedipine (1 μM), GdCl_3 _(1 μM), SKF96365 (7.5 μM), Cyclosporin A (0.03 mg/mL) and EDTA (2 mM), respectively. n = 3, ^### ^*P *< 0.001 vs. hypoxia basal, * *P *< 0.05 vs. hypoxia control, ***P < 0.001 vs. hypoxia control.

Firstly, the mitogenic effect of hypoxia on human PASMC was tested. Cell proliferation was quantified by MTT assay. Hypoxia (3% O_2_) improved cell proliferation significantly (Fig. 1A and 1B). The effect of SOC/[Ca^2+^]_i _in this process was studied to clarify the mechanism of hypoxia-induced PASMC proliferation. Blocking SOC by SKF (7.5 μM) and GdCl_3 _(1 μM, a non-selective cation channel blocker) blocked hypoxia-induced PASMC proliferation. Though SK(7.5 μM) also inhibit cell proliferation under normoxia, the inhibitory efficiency on hypoxia group was significantly greater than that on normoxia group. Nifedipine (1 μM, blocker of VDCC) had no effect on hypoxia-induced cell proliferation. These data suggested that sustained entry of extracellular Ca^2+ ^via SOC is the main pathway of maintaining the high [Ca^2+^]_i _in PASMC. Solvents (DMSO and ethanol) had no obvious effect on cell growth (data not shown).

**Figure 2:**
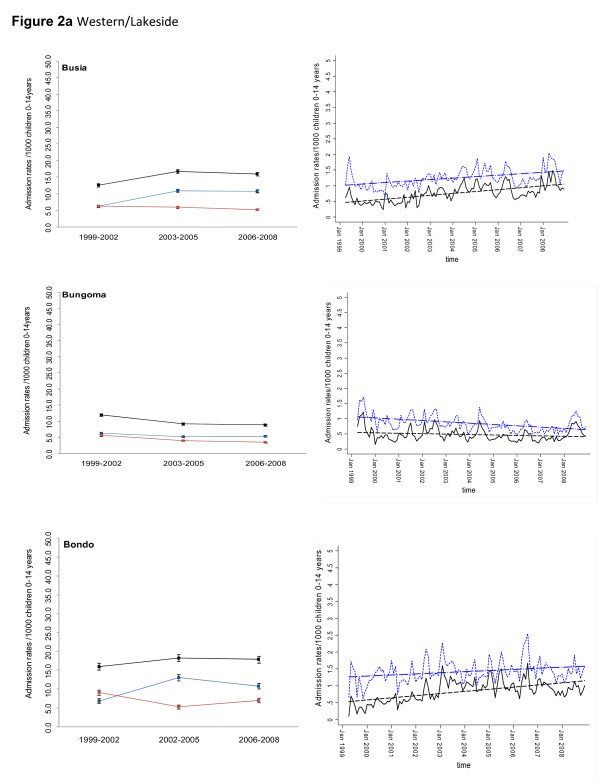
**Inhibitory effect of sildenafil on hypoxia-induced human PASMC proliferation**. Human PASMC were cultured with SMBM (2% FBS) in normoxia or hypoxia in the presence of different concentrations of sildenafil (0 nM, 10 nM, 50 nM, 100 nM) for 72 h. A: Cell viability was measured by MTT. n = 5, ^## ^*P *< 0.01 vs. normoxia, * *P *< 0.05 vs. hypoxia + 0 nM sildenafil. B: 4',6-diamidino-2-phenylindole (DAPI) staining of human PASMC under normoxia or hypoxia with sildenafil (100 nM) for 72 h. a: Image of DAPI stained human PASMC nuclear. b: Summarized data of DAPI stained cell numbers (the average of 3 high power field in every slide).^## ^*P *< 0.01 vs. normoxia, ** *P *< 0.01 vs. hypoxia.

We studied the anti-proliferative effect of sildenafil on hypoxia-induced PASMC proliferation. Sildenafil inhibited the hypoxia-induced increases in cell viability in a dose-dependent manner (Fig. 2A). Sildenafil at 100 nM inhibited the hypoxia-induced increase in PASMC (viability approximately to the control level). This concentration was therefore subsequently used as the inhibitory dose subsequently as previously described [5,24].

### Sildenafil inhibits hypoxia-mediated enhancement of SOC/[Ca^2+^]_i _in human PASMC

**Figure 3:**
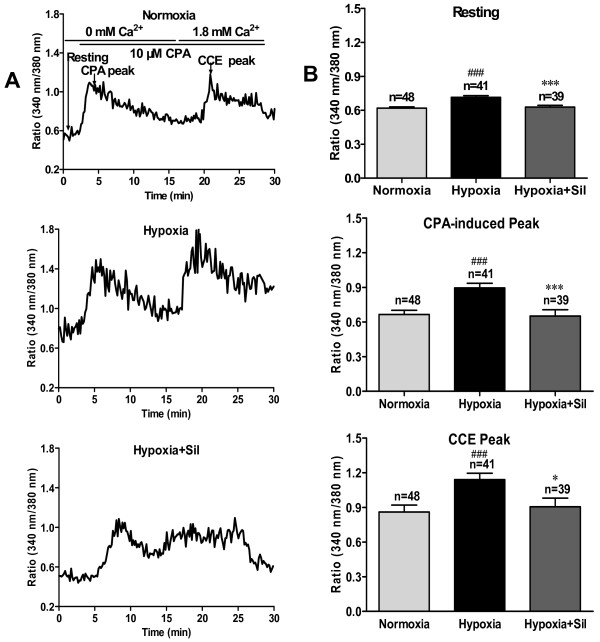
**Inhibitory effect of sildenafil on hypoxia-induced enhancement of resting [Ca^2+^]_i_, CPA-induced ER release and SOC-mediated Ca^2+ ^influx**. A: Representative records of resting [Ca^2+^]_i_, cyclopiazonic acid (CPA)-induced ER Ca^2+ ^release and SOC-mediated Ca^2+ ^entry upon changing perfusion from Ca^2+^-free PSS to 1.8 mM Ca^2+ ^PSS were measured in different groups. B: The statistic data of resting [Ca^2+^]_i_, CPA-inducted ER release, and CCE are expressed as the mean ± SEM. ^### ^*P *< 0.001 vs. normoxia, * *P *< 0.05 vs. hypoxia, *** *P *< 0.001 vs. hypoxia.

Hypoxia-induced PASMC proliferation is associated with extracellular Ca^2+ ^influx through SOC, we investigated if the anti-proliferative effects of sildenafil was related to the changes of [Ca^2+^]_i _and SOCE evoked by hypoxia. Perfusion with Ca^2+^-free PSS containing 10 μM CPA (blocker of ER Ca^2+^-Mg^2+^ATPase) triggered a transient rise in [Ca^2+^]_i _in human PASMC (Fig. 3A) due to leakage of Ca^2+ ^from the ER to the cytosol. The CPA-induced transient rise in [Ca^2+^]_i _declined back to baseline level after 5-10 min as the ER Ca^2+ ^was depleted. Under these conditions, subsequent restoration of extracellular [Ca^2+^]_i _to 1.8 mM (normal PSS) induced a rise in [Ca^2+^]_i _that was obviously due to SOCE (Fig. 3A). Hypoxia induced a significant increase in the resting level of [Ca^2+^]_i _(from 0.619 ± 0.011 to 0.715 ± 0.015, *P *< 0.001), the CPA-induced [Ca^2+^]_i _transient rise due to Ca^2+ ^release from the SR (from 0.666 ± 0.036 to 0.896 ± 0.040, *P *< 0.001) and the peak in [Ca^2+^]_i _due to SOCE (from 0.860 ± 0.059 to 1.144 ± 0.054, *P *< 0.001) in human PASMC compared with normoxia group (Fig. 3B). Sildenafil (100 nM) markedly inhibited hypoxia-mediated increase in resting [Ca^2+^]_i_, CPA-induced peak [Ca^2+^]_i _and CCE (resting [Ca^2+^]_i _from 0.715 ± 0.015 to 0.629 ± 0.015, *P *< 0.001; CPA-induced peak from 0.896 ± 0.040 to 0.652 ± 0.055, *P *< 0.001; SOCE from 1.144 ± 0.054 to 0.905 ± 0.075, *P *< 0.05). These results gave evidence that sildenafil may exert its anti-proliferative effect by inhibiting the activated SOC/[Ca^2+^]_i _pathway under hypoxia exposure.

### Sildenafil inhibits hypoxia-induced up-regulation of TRPC1 expression in human PASMC

TRPC-encoded proteins may be involved in the molecular identity of SOC [25]. Inhibition of TRPC channel expression can inhibit PASMC proliferation [26]. TRPC1 protein is a subunit of SOC in human PASMC, and its activity and expression can affect SOC-mediated Ca^2+ ^influx [27].

**Figure 4:**
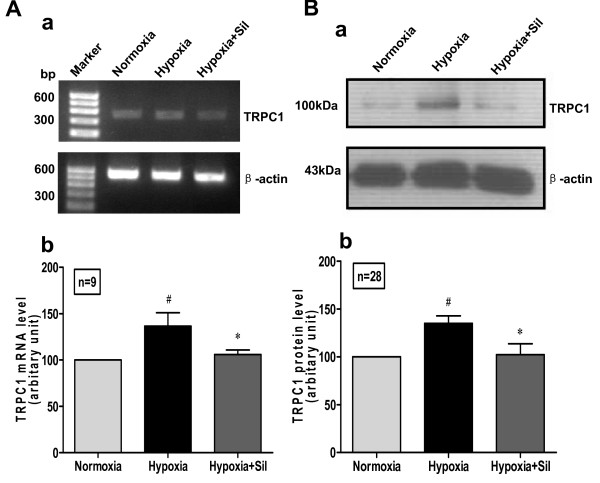
**Inhibitory effect of sildenafil on hypoxia-induced TRPC1 up-regulation**. Human PASMC were cultured with SMBM (2% FBS) under normoxia or hypoxia in the presence or absence of sildenafil (100 nM) for 72 h. A: RT-PCR results. a: PCR amplified products are displayed for TRPC1(372 bp) and β-actin (539 bp). b: Data normalized to the amount of β-actin are expressed as mean ± SEM. n = 9, #*P *< 0.05 vs. normoxia, *P < 0.05 vs. hypoxia. B: Western Blotting results. a: Western boltting results are displayed for TRPC1 (87 kDa) and β-actin (42 kDa). b: Data normalized to the amount of β-actin are expressed as means ± SEM. n = 28, ^#^*P *< 0.05 vs. normoxia, *P < 0.05 vs. hypoxia.

We examined if the anti-proliferation effect of sildenafil is related to the SOC expression. Sildenafil significantly inhibited the up-regulated mRNA and protein expression level of TRPC1 by hypoxia stimulus (Fig. 4). These data lead us to hypothesize that inhibition of TRPC1 expression (at the transcription and translation level) and attenuation of SOC-mediated Ca^2+ ^influx may be the potential pathway mechanism involved in the anti-proliferative effect of sildenafil.

### Sildenafil and SKF inhibited hypoxia induced NFATc3 nuclear translocation

**Figure 5:**
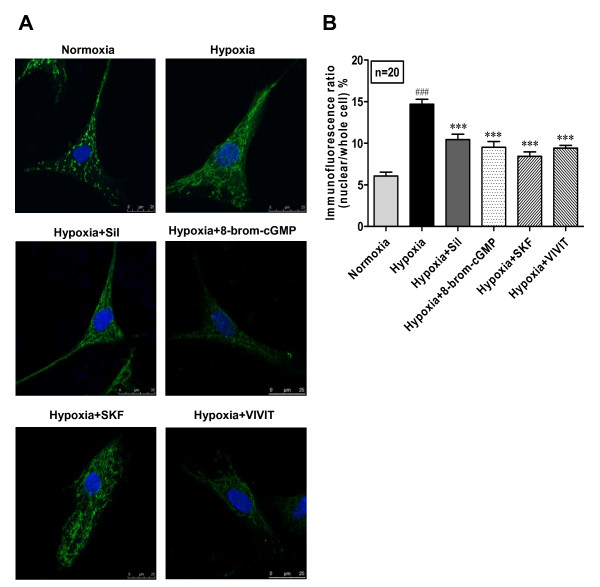
**Sildenafil inhibits hypoxia-induced nuclear translocation of NFATc3 in cultured human PASMC**. Human PASMC were cultured with SMBM (2% FBS) under nomoxia or hypoxia (3% O_2_) in the presence of sildenafil (100 nM), 8-brom-cGMP (100 μM), SKF96365 (7.5 μM) or VIVIT (4 μM) respectively for 72 h. NFATc3 was determined by confocal microscopy of immunofluorescence. The primary antibody of NFATc3 was detected with Rhodamine (TRITC)-conjugated AffiniPure Goat Anti-mouse IgG (green). Slides were counterstained with nuclei dye hoechest33258 (blue). A: Immunofluorescence image of NFATc3 in human PASMC (×1000). B: The nuclear translocation of NFATc3 was calculated by comparing the ratio of nuclear NFATc3 immunofluorescence/total NFATc3 immunofluorescence. n = 20, ### P < 0.001 vs. normoxia, *** P < 0.001 vs. hypoxia.

Increased [Ca^2+^]_i _activates calcineurin which dephosphorylates cytoplasmic NFAT, allowing its entry to the nucleus where it forms complexes with other transcription factors and regulates gene transcriptions [28]. We demonstrated that [Ca^2+^]_i _was significantly increased in hypoxic PASMC. We assessed if this hypoxia-induced [Ca^2+^]_i _increase through SOC could mediate NFAT nuclear translocation. The results show that hypoxia induced significant nuclear translocation of NFATc3 (Fig. 5A), which was inhibited not only by the SOC blocker SKF, but also by sildenafil. To confirm the influence of cGMP on NFATc3 activation, we observed the effect of 8-brom-cGMP. Similar to sildenafil, 8-brom-cGMP also showed inhibitory effect on NFATc3 nuclear translocation (Fig. 5B). These results suggest that hypoxia-induced NFAT nuclear translocation is dependent on Ca^2+ ^influx through SOC. The antiproliferative property of sildenafil on PASMC may related to the decreased TRPC1 expression which attenuates SOC-mediated Ca^2+ ^influx, calcineurin activity and NFAT nuclear translocation.

### NFAT nuclear translocation is involved in hypoxia-induced TRPC1 up-regulation and human PASMC proliferation

**Figure 6:**
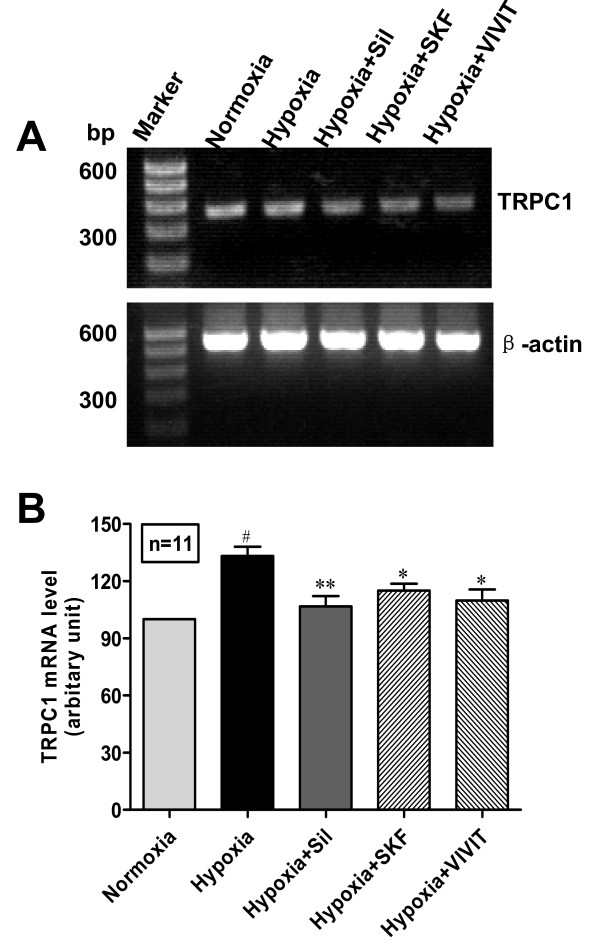
**NFAT inhibitor, VIVIT inhibits hypoxia-induced TRPC1 mRNA up-regulation**. Human PASMC were cultured with SMBM (2% FBS) under nomoxia or hypoxia (3% O_2_) in the presence of sildenafil (100 nM), SKF96365 (7.5 μM) or VIVIT (4 μM) respectively for 72 h. A: PCR amplified products are displayed for TRPC1 (372 bp) and β-actin (539 bp). B: Datanormalized to the amount of β-actin are expressed as mean ± SEM. n = 11, ^#^*P *< 0.05 vs. normoxia, *P < 0.05 vs. hypoxia, **P < 0.01 vs. hypoxia.

**Figure 7:**
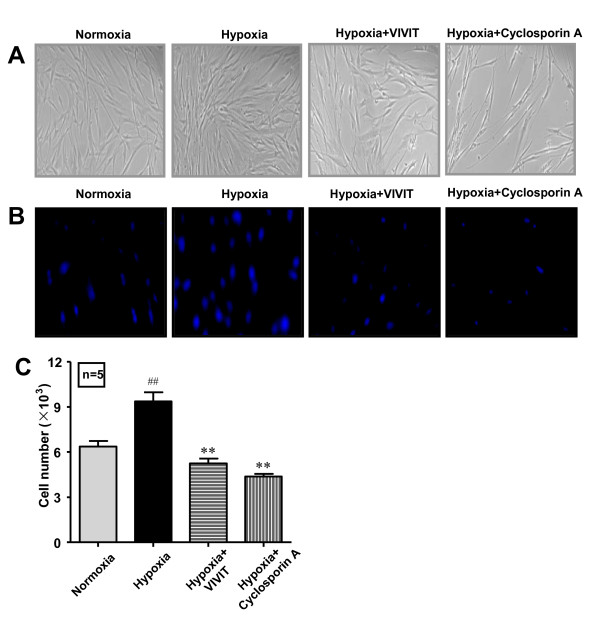
**NFAT inhibitors, VIVIT and Cyclosporin A inhibitshypoxia-induced human PASMC proliferation**. Cell proliferation wasdetected before (Basal) and after 72 h incubation under normoxia, hypoxia, hypoxia plus VIVIT (4 μM) and hypoxia plus Cyclosporin A (0.03 mg/mL) respectively. A: Phase contrast image of cultured human PASMC (×200). B: Image of DAPI stained human PASMC. C: Cell number was determined by cell counting. n = 5, ^## ^*P *< 0.01 vs. normoxia, ** *P *< 0.01 vs. hypoxia.

The effects of a direct and specific inhibitor of NFAT (VIVIT) and an indirect inhibitor of NFAT (Cyclosporin A) on hypoxia-induced TRPC1 up-regulation and human PASMC proliferation were examined. As shown in Fig. 6 and Fig. 7, VIVIT and Cyclosporin A inhibited hypoxia-induced TRPC1 up-regulation, as well as human PASMC proliferation. No significant influence of solvent control ethanol on human PASMC proliferation was detected (data not shown).

## Discussion

In the present study we demonstrated: (a) Up-regulation of TRPC1 expression, enhancement of SOC-mediated Ca^2+ ^influx and increase in [Ca^2+^]_i _are involved in hypoxia-induced human PASMC proliferation. (b) Potentiation of [Ca^2+^]_i _resulting from enhancement of SOC leads to nuclear translocation of NFATc3. (c) NFATc3 nuclear translocation is involved in hypoxia-induced human PASMC proliferation; (d) Inhibiting NFAT nuclear translocation reduces TRPC1 expression in human PASMC. (e) Anti-proliferative effects of sildenafil is related to the SOC/Ca^2+^/NFAT pathway. PAH is a disease of progressive vascular remodeling of the small pulmonary arteries (<500 μM in diameter), which results in a progressive increase in pulmonary vascular resistance and, eventually, right ventricular failure and death [29]. The typical pathological changes include muscularization and thickening of pre-capillary pulmonary arteries, intimal proliferation, obliterative lesions, and thrombosis *in situ *[29]. Pulmonary vascular remodeling is characterized by uncontrolled and inappropriate proliferation of PASMC [17], which is closely related to the malfunction of endothelin, NO/cGMP and prostacyclin pathways.

The NO/cGMP axis is one of the major target for PAH treatment. PDE5 as a major cGMP-degrading phosphodiesterase in the pulmonary vasculature, is up-regulated in PAH [30-32], and may contribute to the impaired vasodilator responses in the hypoxic lung. Sildenafil is an orally active, potent and selective inhibitor of PDE5 that can elevate the level of intracellular cGMP level by inhibiting PDE5 activity and cGMP breakdown. Animal studies have demonstrated that oral treatment with sildenafil significantly reduces neomuscularization in hypoxia and monocrotaline models of pulmonary hypertension [2,33]. Several studies concerning the remodeling process revealed more promising options for therapy in addition to the NO/cGMP pathway [34-37]. Sildenafil has been shown recently that it can act through preventing Ras homolog gene family, member A (RhoA) expression[37]. We have shown in the recent study that sildenafil can inhibit ET-1 induced PASMC proliferation by decreasing TRPC1 expression, [Ca^2+^]_i _and SOC-mediated Ca^2+ ^influx [36].

Previous researches suggested that cGMP/PKG pathway had effect on TRP activity. PKG could directly phosphorylate TRPC3 channels and abolish TRPC3 mediated store-operated Ca^2+ ^influx[38]. TRPC6 channels can be negatively regulated by the NO/cGMP/PKG pathway in smooth muscle cells[39]. NO contributes to the vasorelaxation by inhibition of La^3+^-sensitive channels consistent with TRPC1/C3[40]. In addition, cGMP/PKG was reported to have a role in the activity of transcription factors, such as NFAT, which can regulate TRPC gene expression[41]. Our results suggested that 8-brom-cGMP could inhibit the translocation of NFAT, and these data provided evidence that cGMP may be involved in SOC/Ca^2+^/NFAT pathway, but the exact mechanism needs further research.

Intracellular Ca^2+^, as an essential factor that participates in cell cycle and promotes transcription factor binding activity with mitogenic genes, is intimately involved in cell proliferation [42]. [Ca^2+^]_i _is reported higher in the PASMC of PAH patients. Hypoxia-induced PASMC proliferation was accompanied by a significant increase in resting [Ca^2+^]_i_, calcium release from the ER, and SOC-mediated Ca^2+ ^influx in cytoplasm (Fig. 3). Chelating extracellular Ca^2+ ^or blockade of Ca^2+ ^influx via SOC inhibited hypoxia-induced PASMC proliferation, but VDCC played little part in hypoxia-induced PASMC proliferation (Fig. 1). TRPC1 has been demonstrated to be involved in the formation of SOC that contributes to the development of cardiac hypertrophy. Up-regulation of TRPC1 and increase in SOC-mediated Ca^2+ ^influx were observed in cardiomyocytes with chronic treatment of G-protein coupled receptors (GPCR) agonists such as ET-1, angiotensin-II, and phenylephrine [43]. Up-regulated TRPC1 gene expression in human PASMC would therefore be predicted to increase the number of functional SOC, enhance vasoconstrictor and mitogen-mediated increases in [Ca^2+^]_i_, stimulate vasoconstriction, and promote cell growth. We demonstrated that hypoxia promoted TRPC1 expression on the genetic and protein level which might be responsible for the enhancement of SOC-mediated Ca^2+ ^influx.

An important function of Ca^2+ ^is its role in regulation of gene expression. One mechanism by which the Ca^2+ ^signal can be translated into a change in gene activity is the calcineurin-mediated activation of NFAT [44]. NFAT has been described as a signal integrator of Ca^2+ ^signal and other signaling pathways with induction of a specific genetic program. In T-cells, depletion of intracellular Ca^2+ ^stores resulted in persistent Ca^2+ ^influx via SOC, a process that was necessary to maintain NFAT proteins in the nucleus [45].

The effect of Ca^2+^/calcineurin/NFAT pathway has been extensively investigated in cardiac hypertrophy [10,46]. A model has been proposed whereby calcineurin transduces the Ca^2+^signal generated by sarcomeric dysfunction, mechanical load, or chemical agonists through dephosphorylation and activation of the NFAT transcription factor. The nuclear NFAT protein would then cooperate with cardiomyocyte-expressed transcription factors to initiate the hypertrophic gene expression program [47]. Given the recurrent theme of NFAT regulation of hypertrophic tissue responses in the literature, we speculate a similar role for NFAT in vascular smooth cell hypertrophy and proliferation associated with PAH. Frutos et al [12] demonstrated that NFATc3 was expressed in the mouse pulmonary artery (PA). Chronic hypoxia increased NFATc3 transcriptional activity and nuclear translocation. NFATc3 may mediate chronic hypoxia-induced PA remodeling with α-actin up-regulation. Bonnet et al suggested that NFAT activation contributed significantly to voltage-gated potassium channel 1.5 (Kv1.5) down-regulation, bcl-2 up-regulation and mitochondrial hyperpolarization, all of which contributed to remodeling of the pulmonary artery [13]. The present study further demonstrates that hypoxia induces NFAT nuclear translocation via the up-regulation of TRPC1, increase in SOC-mediated Ca^2+ ^influx and elevation of [Ca^2+^]_i_. It has been showed that the TRPC1 promoter has one binding sequence with NFAT. Conceivably, once activated, NFAT might stimulate TRPC1 expression through a positive feedback mechanism. The results in this experiment showed that blocking NFAT nuclear translocation inhibited hypoxia-induced TRPC1 expression. These results suggested that the Ca^2+^/calcineurin/NFAT pathway especially this positive feedback mechanism could feasibly stimulate the development of hypoxia-induced PASMC proliferation.

Our experiments demonstrated that sildenafil not only attenuated hypoxia-induced elevation of TRPC1 expression, enhancement of SOC function and increase in [Ca^2+^]_i_, but also inhibited NFAT nuclear translocation. The results indicated that therapeutic effects (vasodilation/antiproliferation) of sildenafil was involved in inhibition of the SOC/Ca^2+^/NFAT pathway. Additionally, activation PKG pathway by sildenafil and 8-brom-cGMP inhibited NFAT nuclear translocation. We speculate that the sildenafil effect in PAH therapy may act through multiple downstream signaling pathways and target the progression of pulmonary vascular remodeling in PAH. Novel strategies involving NFAT inhibition can be useful for PAH treatment.

## Competing interests

The authors declare that they have no competing interests.

## Authors' contributions

CW carried out Western blotting, Ca^2+ ^imaging and the design of the study. JFL, JL, JW and YXW carried out immunofluorescence and RT-PCR as well as MTT experiment. LZ, JW and CW prepared the manuscript. All authors read and approved the final manuscript.

## References

[B1] Humbert M Sitbon O Simonneau G Treatment of pulmonary arterial hypertension N Engl J Med 2004 351 14 1425 1436 10.1056/NEJMra040291 15459304

[B2] Sebkhi A Strange JW Phillips SC Wharton J Wilkins MR Phosphodiesterase type 5 as a target for the treatment of hypoxia-induced pulmonary hypertension Circulation 2003 107 25 3230 3235 10.1161/01.CIR.0000074226.20466.B1 12796132

[B3] Mathai SC Girgis RE Fisher MR Champion HC Housten-Harris T Zaiman A Hassoun PM Addition of sildenafil to bosentan monotherapy in pulmonary arterial hypertension Eur Respir J 2007 29 3 469 475 10.1183/09031936.00081706 17079256

[B4] Kirsch M Kemp-Harper B Weissmann N Grimminger F Schmidt HH Sildenafil in hypoxic pulmonary hypertension potentiates a compensatory up-regulation of NO-cGMP signaling Faseb J 2008 22 1 30 40 10.1096/fj.06-7526com 17679609

[B5] Tantini B Manes A Fiumana E Pignatti C Guarnieri C Zannoli R Branzi A Galie N Antiproliferative effect of sildenafil on human pulmonary artery smooth muscle cells Basic Res Cardiol 2005 100 2 131 138 10.1007/s00395-004-0504-5 15739122

[B6] Ikeda F Nishimura R Matsubara T Hata K Reddy SV Yoneda T Activation of NFAT signal in vivo leads to osteopenia associated with increased osteoclastogenesis and bone-resorbing activity J Immunol 2006 177 4 2384 2390 16888000 10.4049/jimmunol.177.4.2384

[B7] Heit JJ Apelqvist AA Gu X Winslow MM Neilson JR Crabtree GR Kim SK Calcineurin/NFAT signalling regulates pancreatic beta-cell growth and function Nature 2006 443 7109 345 349 10.1038/nature05097 16988714

[B8] Nilsson LM Sun ZW Nilsson J Nordstrom I Chen YW Molkentin JD Wide-Swensson D Hellstrand P Lydrup ML Gomez MF Novel blocker of NFAT activation inhibits IL-6 production in human myometrial arteries and reduces vascular smooth muscle cell proliferation Am J Physiol Cell Physiol 2007 292 3 C1167 1178 10.1152/ajpcell.00590.2005 17079331

[B9] Jabr RI Wilson AJ Riddervold MH Jenkins AH Perrino BA Clapp LH Nuclear translocation of calcineurin Abeta but not calcineurin Aalpha by platelet-derived growth factor in rat aortic smooth muscle Am J Physiol Cell Physiol 2007 292 6 C2213 2225 10.1152/ajpcell.00139.2005 17303652

[B10] Nishida M Onohara N Sato Y Suda R Ogushi M Tanabe S Inoue R Mori Y Kurose H Galpha12/13-mediated up-regulation of TRPC6 negatively regulates endothelin-1-induced cardiac myofibroblast formation and collagen synthesis through nuclear factor of activated T cells activation J Biol Chem 2007 282 32 23117 23128 10.1074/jbc.M611780200 17533154

[B11] Friday BB Pavlath GK A calcineurin- and NFAT-dependent pathway regulates Myf5 gene expression in skeletal muscle reserve cells J Cell Sci 2001 114 Pt 2 303 310 11148132 10.1242/jcs.114.2.303

[B12] de Frutos S Spangler R Alo D Bosc LV NFATc3 mediates chronic hypoxia-induced pulmonary arterial remodeling with alpha-actin up-regulation J Biol Chem 2007 282 20 15081 15089 10.1074/jbc.M702679200 17403661 PMC2754407

[B13] Bonnet S Rochefort G Sutendra G Archer SL Haromy A Webster L Hashimoto K Bonnet SN Michelakis ED The nuclear factor of activated T cells in pulmonary arterial hypertension can be therapeutically targeted Proc Natl Acad Sci USA 2007 104 27 11418 11423 10.1073/pnas.0610467104 17596340 PMC1903339

[B14] Rao A Luo C Hogan PG Transcription factors of the NFAT family: regulation and function Annul Rev Immunol 1997 15 707 747 10.1146/annurev.immunol.15.1.707 9143705

[B15] Hogan PG Chen L Nardone J Rao A Transcriptional regulation by calcium, calcineurin, and NFAT Genes Dev 2003 17 18 2205 2232 10.1101/gad.1102703 12975316

[B16] Parekh AB Putney JW Jr Store-operated calcium channels Physiol Rev 2005 85 2 757 810 10.1152/physrev.00057.2003 15788710

[B17] Remillard CV Yuan JX TRP channels, CCE, and the pulmonary vascular smooth muscle Microcirculation 2006 13 8 671 692 10.1080/10739680600930313 17085427

[B18] Zhang S Patel HH Murray F Remillard CV Schach C Thistlethwaite PA Insel PA Yuan JX Pulmonary artery smooth muscle cells from normal subjects and IPAH patients show divergent cAMP-mediated effects on TRPC expression and capacitative Ca^2+ ^entry Am J Physio Lung Cell Mol Physiol 2007 292 5 L1202 1210 10.1152/ajplung.00214.2006 17189322

[B19] Wang J Weigand L Lu W Sylvester JT Semenza GL Shimoda LA Hypoxia inducible factor 1 mediates hypoxia-induced TRPC expression and elevated intracellular Ca^2+ ^in pulmonary arterial smooth muscle cells Circ Res 2006 98 12 1528 1537 10.1161/01.RES.0000227551.68124.98 16709899

[B20] Fantozzi I Zhang S Platoshyn O Remillard CV Cowling RT Yuan JX Hypoxia increases AP-1 binding activity by enhancing capacitative Ca^2+ ^entry in human pulmonary artery endothelial cells Am J Physiol Lung Cell Mol Physiol 2003 285 6 L1233 1245 12909593 10.1152/ajplung.00445.2002

[B21] Weissmann N Dietrich A Fuchs B Kalwa H Ay M Dumitrascu R Olschewski A Storch U Mederosy Schnitzler M Ghofrani HA Schermuly RT Pinkenburg O Seeger W Grimminger F Gudermann T Classical transient receptor potential channel 6 (TRPC6) is essential for hypoxic pulmonary vasoconstriction and alveolar gas exchange Proc Natl Acad Sci USA 2006 103 50 19093 19098 10.1073/pnas.0606728103 17142322 PMC1748182

[B22] Kunichika N Landsberg JW Yu Y Kunichika H Thistlethwaite PA Rubin LJ Yuan JX Bosentan inhibits transient receptor potential channel expression in pulmonary vascular myocytes Am J Respir Crit Care Med 2004 170 10 1101 1107 10.1164/rccm.200312-1668OC 15317671

[B23] Grynkiewicz G Poenie M Tsien RY A new generation of Ca^2+ ^indicators with greatly improved fluorescence properties J Biol Chem 1985 260 6 3440 3450 3838314

[B24] Pauvert O Bonnet S Rousseau E Marthan R Savineau JP Sildenafil alters calcium signaling and vascular tone in pulmonary arteries from chronically hypoxic rats Am J Physiol Lung Cell Mol Physiol 2004 287 3 L577 583 10.1152/ajplung.00449.2003 15155272

[B25] Vannier B Peyton M Boulay G Brown D Qin N Jiang M Zhu X Birnbaumer L Mouse trp2, the homologue of the human trpc2 pseudogene, encodes mTrp2, a store depletion-activated capacitative Ca^2+ ^entry channel Proc Natl Acad Sci USA 1999 96 5 2060 2064 10.1073/pnas.96.5.2060 10051594 PMC26736

[B26] Sweeney M Yu Y Platoshyn O Zhang S McDaniel SS Yuan JX Inhibition of endogenous TRP1 decreases capacitative Ca^2+ ^entry and attenuates pulmonary artery smooth muscle cell proliferation Am J Physiol Lung Cell Mol Physiol 2002 283 1 L144 155 12060571 10.1152/ajplung.00412.2001

[B27] Golovina VA Platoshyn O Bailey CL Wang J Limsuwan A Sweeney M Rubin LJ Yuan JX Upregulated TRP and enhanced capacitative Ca^2+ ^entry in human pulmonary artery myocytes during proliferation Am J Physiol Heart Circ Physiol 2001 280 2 H746 755 11158974 10.1152/ajpheart.2001.280.2.H746

[B28] Macian F NFAT proteins: key regulators of T-cell development and function Nat Rev Immunol 2005 5 6 472 484 10.1038/nri1632 15928679

[B29] Runo JR Loyd JE Primary pulmonary hypertension Lancet 2003 361 9368 1533 1544 10.1016/S0140-6736(03)13167-4 12737878

[B30] Black SM Sanchez LS Mata-Greenwood E Bekker JM Steinhorn RH Fineman JR sGC and PDE5 are elevated in lambs with increasedpulmonary blood flow and pulmonary hypertension Am J Physiol Lung Cell Mol Physiol 2001 281 5 L1051 1057 11597895 10.1152/ajplung.2001.281.5.L1051

[B31] Rondelet B Kerbaul F Van Beneden R Motte S Fesler P Hubloue I Remmelink M Brimioulle S Salmon I Ketelslegers JM Signaling molecules in overcirculation-induced pulmonary hypertension in piglets: effects of sildenafil therapy Circulation 2004 110 15 2220 2225 10.1161/01.CIR.0000143836.40431.F5 15466636

[B32] Wharton J Strange JW Moller GM Growcott EJ Ren X Franklyn AP Phillips SC Wilkins MR Antiproliferative effects of phosphodiesterase type 5 inhibition in human pulmonary artery cells Am J Respir Crit Care Med 2005 172 1 105 113 10.1164/rccm.200411-1587OC 15817798

[B33] Schermuly RT Kreisselmeier KP Ghofrani HA Yilmaz H Butrous G Ermert L Ermert M Weissmann N Rose F Guenther A Walmrath D Seeger W Grimminger F Chronic sildenafil treatment inhibits monocrotaline-induced pulmonary hypertension in rats Am J Respir Crit Care Med 2004 169 1 39 45 10.1164/rccm.200302-282OC 12958054

[B34] Schermuly RT Dony E Ghofrani HA Pullamsetti S Savai R Roth M Sydykov A Lai YJ Weissmann N Seeger W Grimminger F Reversal of experimental pulmonary hypertension by PDGF inhibition J Clin Invest 2005 115 10 2811 2821 10.1172/JCI24838 16200212 PMC1236676

[B35] Krick S Hanze J Eul B Savai R Seay U Grimminger F Lohmeyer J Klepetko W Seeger W Rose F Hypoxia-driven proliferation of human pulmonary artery fibroblasts: cross-talk between HIF-1alpha and an autocrine angiotensin system Faseb J 2005 19 7 857 859 15718424 10.1096/fj.04-2890fje

[B36] Wang C Wang J Zhao L Wang YX Liu J Shi LP Xu M Wang C Sildenafil inhibits human pulmonary artery smooth muscle cell proliferation by decreasing capacitative Ca^2+ ^entry J Pharmacol Sci 2008 108 1 71 78 10.1254/jphs.08069FP 18818482

[B37] Fukumoto Y Tawara S Shimokawa H Recent progress in the treatment of pulmonary arterial hypertension: expectation for rho-kinase inhibitors Tohoku J Exp Med 2007 211 4 309 320 10.1620/tjem.211.309 17409670

[B38] Kwan HY Huang Y Yao X Regulation of canonical transient receptor potential isoform 3 (TRPC3) channel by protein kinase G Proc Natl Acad Sci USA 2004 101 8 2625 2630 10.1073/pnas.0304471101 14983059 PMC357000

[B39] Takahashi S Lin H Geshi N Mori Y Kawarabayashi Y Takami N Mori MX Honda A Inoue R Nitric oxide-cGMP-protein kinase G pathway negatively regulates vascular transient receptor potential channel TRPC6 J Physiol 2008 586 Pt 17 4209 4223 10.1113/jphysiol.2008.156083 18617565 PMC2652196

[B40] Chen J Crossland RF Noorani MM Marrelli SP Inhibition of TRPC1/TRPC3 by PKG contributes to NO-mediated vasorelaxation Am J Physiol Heart Circ Physiol 2009 297 1 H417 424 10.1152/ajpheart.01130.2008 19502552 PMC2711742

[B41] Fiedler B Lohmann SM Smolenski A Linnemuller S Pieske B Schroder F Molkentin JD Drexler H Wollert KC Inhibition of calcineurin-NFAT hypertrophy signaling by cGMP-dependent protein kinase type I in cardiac myocytes Proc Natl Acad Sci USA 2002 99 17 11363 11368 10.1073/pnas.162100799 12177418 PMC123262

[B42] Hardingham GE Chawla S Johnson CM Bading H Distinct functions of nuclear and cytoplasmic calcium in the control of gene expression Nature 1997 385 6613 260 265 10.1038/385260a0 9000075

[B43] Ohba T Watanabe H Murakami M Takahashi Y Iino K Kuromitsu S Mori Y Ono K Iijima T Ito H Upregulation of TRPC1 in thedevelopment of cardiac hypertrophy J Mol Cell Cardiol 2007 42 3 498 507 10.1016/j.yjmcc.2006.10.020 17174323

[B44] Hill-Eubanks DC Gomez MF Stevenson AS Nelson MT NFAT regulation in smooth muscle Trends Cardiovasc Med 2003 13 2 56 62 10.1016/S1050-1738(02)00212-8 12586440

[B45] Gwack Y Feske S Srikanth S Hogan PG Rao A Signalling to transcription: store-operated Ca^2+ ^entry and NFAT activation in lymphocytes Cell calcium 2007 42 2 145 156 10.1016/j.ceca.2007.03.007 17572487

[B46] Chien KR Zhu H Knowlton KU Miller-Hance W van-Bilsen M O'Brien TX Evans SM Transcriptional regulation during cardiac growth and development Annu Rev Physiol 1993 55 77 95 10.1146/annurev.ph.55.030193.000453 8466192

[B47] Sadoshima J Izumo S The cellular and molecular response of cardiac myocytes to mechanical stress Annu Rev Physiol 1997 59 551 571 10.1146/annurev.physiol.59.1.551 9074777

